# Genetic Diversity and Health Properties of Polyphenols in Potato

**DOI:** 10.3390/antiox11040603

**Published:** 2022-03-22

**Authors:** Haroon Rasheed, Daraz Ahmad, Jinsong Bao

**Affiliations:** Key Laboratory of Nuclear Agricultural Sciences of Ministry of Agriculture and Zhejiang Province, Institute of Nuclear Agricultural Sciences, College of Agriculture and Biotechnology, Zhejiang University, Hangzhou 310058, China; haroonzju@zju.edu.cn (H.R.); 11916123@zju.edu.cn (D.A.)

**Keywords:** potato, polyphenol, health properties

## Abstract

Polyphenol is one of the most essential phytochemicals with various health benefits. Potato (*Solanum tuberosum* L.) is known as a potential source of polyphenols, and also has health benefits in which phenolic acids, such as chlorogenic, ferulic acid, caffeic acid, and flavonoids, such as anthocyanins, sustainably play the most significant role. Almost every polyphenol contributes to various biological activities. In this review, we collected comprehensive information concerning the diversity of polyphenols in potatoes, and the effects of post-harvest processing and different cooking methods on the bioavailability of polyphenols. To achieve maximum health benefits, the selection of potato cultivars is necessary by choosing their colors, but various cooking methods are also very important in obtaining the maximum concentration of polyphenolic compounds. The health properties including major biological activities of polyphenols, such as antioxidant activity, anticarcinogenic activity, anti-inflammatory activity, anti-obesity activity, and antidiabetic activity, have also been summarized. All these biological activities of polyphenols in potatoes might be helpful for breeders in the design of new varieties with many health benefits, and are expected to play a vital role in both pharmaceutical and nutraceutical industries.

## 1. Introduction

Potatoes (*Solanum tuberosum* L.) are the fourth most important and staple food grown and consumed worldwide [1,2]. It is one of the important sources of the human diet and comprises more than 4000 varieties all over the world. Such a huge variety of potatoes are accepted by the worldwide market due to their high production rate, regular tuber size, and uses for multi-purposes both in food industries and pharmaceutical industries [3]. Mealy potatoes, waxy potatoes, and soggy potatoes are the most common potato cultivars produced in everyday life. Potato is not a key food in many areas of the world, but is widely cultivated with an output of about 374 million tons per year. China is amongst the largest potato production country because of its wide planting area, good adaptability to the environment, and many other aspects [4].

Potato is a carbohydrate-rich food along with a small number of fats which provide about 130 kcal energy to a person per day [5], vitamins, protein, fibers, and many other nutrients. Potatoes are found in various colors due to the presence of carotenoids and anthocyanins, although non-colored potatoes are the most common genotypes [6]. Bioactive compounds such as polyphenols, anthocyanins, and carotenoids are reported extensively in flesh and peel, giving more importance to the potato tubers. Thus, a high intake of potatoes on a daily basis contributes the highest polyphenol content to the diet after oranges and apples [7].

Recent studies demonstrated various applications of natural dye obtained from anthocyanins, which replace artificial colors across multiple food items, including various beneficial effects on human health [8]. Polyphenols, especially some flavonoids like anthocyanins, show a greater capacity to capture free radicals and act as antioxidants, thus helping in the prevention of cardiovascular diseases, cancer, neurodegenerative disorders, and offer a protective effect in diabetic nephropathy induced by inflammation [9].

## 2. Diversity of Polyphenols in Potato

**Figure 1:**
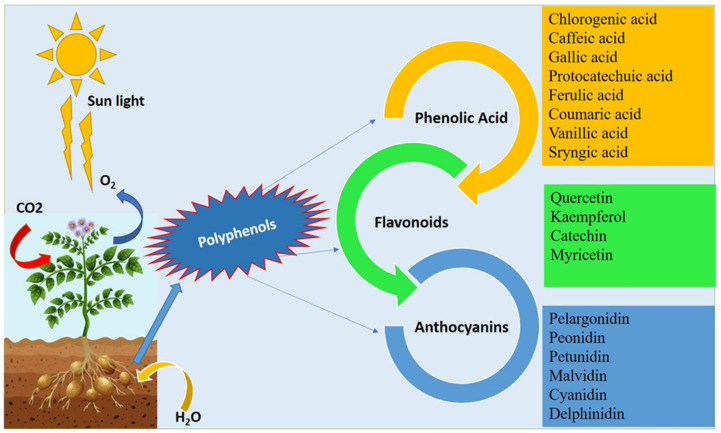
Various polyphenolic compounds present in potato tubers.

Potato polyphenols have gained most of the attention of nutritionists due to their excellent therapeutic and nutritional properties. Potato with colored flesh is a good source of polyphenols which helps in the reduction of various diseases, like cardiovascular diseases, cancer, etc. [10,11]. Potato polyphenols consist of phenolic acids, flavonoids, and anthocyanins. The major phenolic acids reported in potatoes are chlorogenic acid, caffeic acid, ferulic acid, syringic acid, gallic acid, protocatechuic acid, coumaric acid, and vanillic acid; the main flavonoids include kaempferol, quercetin, catechin, and myricetin, while the major anthocyanins are pelargonidin, peonidin, petunidin, malvidin, cyanidin, and delphinidin (Figure 1). The content of phenolic compounds varied significantly depending on the genotype [9,12], the location, and the color of the parenchyma. Polyphenol content in the purple potato cultivar were detected as 209 mg gallic acid equivalent (GAE)/100 g fresh weight (FW) [12]. In a whole potato tuber, the polyphenols ranged from 22.53 to 85.85 mg GAE/100 g FW, while in the flesh it was 20.26 to 63.05 mg GAE/100 g FW [13]. In purple potatoes, the polyphenols ranged from 162.19 to 510.20 mg GAE/100 g dry weight (DW), in red potatoes from 152.40 to 261.49 mg GAE/100 g DW, while in yellow potatoes ranged from 113.37 to 114.63 mg GAE/100 g DW among 20 Andean potato cultivars [14].

### 2.1. Phenolic Acids

Phenolic acids are the most important group of polyphenols present in potatoes [5,14,15,16,17]. Both cinnamic acids and benzoic acids groups are present in potatoes and are synthesized by the shikimate pathway [17]. Cinnamic acid and its derivatives are the most abundantly found phenolic acids in potatoes, along with benzoic acids such as gallic acid and protocatechuic acid. The major phenolic acids in potatoes are chlorogenic acid and caffeic acid followed by protocatechuic acid, p-coumaric acid, ferulic acid, vanillic acid, gallic acid, syringic acid, and salicylic acid [18,19]. The concentration of chlorogenic acid accounted for 35.21 to 81.78% (Table 1) of total free phenolic acid [20]. Potato tubers contain isomers of chlorogenic acid, neochlorogenic acid, cryptochlorogenic acid, and caffeic acids, as well [21]. Potato peels also have a high number of phenolic acids, of which chlorogenic acid is the most abundantly found, while caffeic acid, gallic acid, and protocatechuic acid are present in low amounts [16,22]. Various studies demonstrated that the genetic makeup of a potato variety has more effect on the phenolic content than the environment [23].

Various postharvest processing methods result in the change of polyphenols contents [24,25,26,27,28,29,30,31,32,33,34,35,36]. For example, peeling of blue-fleshed potatoes can decrease phenolic acid content by about 80% while peeling of yellow-fleshed potatoes can decrease about 60% of the phenolic acid content [24]. Such a great reduction in phenolic acid content might be due to the effect of the peeling method, with thicker peeling resulting in higher loss [24]. The content of phenolic acid was decreased during the storage process of fresh-cut potatoes at 10 °C [25], while increased during cold storage at 4 °C [26]. After harvesting, the content of phenolic acid decreased [27]. According to Galani et al. [28], the content of phenolic acid increased with a storage temperature of 15 °C and 4 °C, except for p-coumaric acid, which decreased at low storage temperature in eleven Indian potato varieties assessed for antioxidant activity.

Cooking can reduce phenolic acids; for example, the boiling method can reduce a small number of phenolic acids in potatoes [29]. The contents of soluble polyphenols decreased with boiling and steaming cooking processes [30]. Microwaving and frying may result in the reduction of polyphenol in potato cultivars with different peel colors [29,31,32]. According to Lachman et al. [34], the concentration of chlorogenic acid in tubers is decreased significantly by various cooking methods like boiling, microwaving, and baking. Faller and Fialho reported that microwaving increased the soluble polyphenols by 11%, and boiling, microwaving, and steaming increased hydrolyzable polyphenols by 81.4%, 80.81%, and 22.8%, respectively [30]. In cooked tubers of 113 potato varieties, the concentration of chlorogenic acid ranged from 0.77 to 7.98 mg/100 g fresh weight [21], while in cooked purple potatoes the concentration of chlorogenic acid range from 36.1 to 395.73 mg/100 g fresh weight, and 14.45 to 48.60 mg/100 g fresh weight was recorded for red-fleshed cultivars [35]. Navarre et al. [36] reported that boiling, baking, stir-frying, microwaving, and steaming increased chlorogenic acid concentration. In purple potato cultivars, the chlorogenic contents were increased from 2.14 to 2.92 mg/g DW after baking (Table 1); the uncooked potatoes have a phenolic content of 209 ± 35.7 mg GAE/100 g (fresh weight), whereas, boiling, steaming, microwaving and baking reduce the content to 137.6 ± 0 mg GAE/100 g, 130.4 ± 3.7 mg GAE/100 g, 74.0 ± 1.3 mg GAE/100 g and 38.1 ± 7.5 mg GAE/100 g, respectively [12]. Ezekiel et al. [5] stated that cooking results in limited decreases in phenolic contents. Cooking potatoes with peel may decrease leaching because the fleshy tissues are not directly exposed to water. Based on the information listed above, the variability of polyphenolic contents might be due to different conditions of cooking methods (like method of heat transferring, amount of added water, and time requirement), genotype and varieties, and growing location.

### 2.2. Flavonoids

Potatoes consist of flavonoids such as catechin, quercetin, kaempferol-rutinose and rutin [26]. The concentration of flavonoid is two times higher in red and purple-fleshed potato cultivars as compared to white-fleshed potato cultivars [51]. Flavonols are predominantly found in white and yellow-fleshed potatoes compared to red and purple-fleshed potatoes [52]. Among potato flavonoids, catechin is the most abundant, and ranges from 0–204 mg/100 g dry weight [53]. Kaempferol, quercetin, and myricetin are also found in potatoes, but colored potatoes have double the amount compared to white and yellow potato varieties [53]. Quercetin and kaempferol were detected as 0–4.78 and 0–5.68 mg/100 g of fresh weight, respectively, and they are the most important compounds in Andean potato cultivars [52].

Navarre et al. [36] reported that baking process increased the rutin content from 7.4 to 13.2, and increased kaempferol-rutinose from 7.0 to 17.3 µg/g DW in various purple-fleshed potato cultivars (Table 2). Various cooking methods like boiling, frying, baking, and microwaving significantly decreased the quercetin contents, while a higher level of epicatechin was observed in potatoes due to microwaving [54]. Tudela et al. [29] also observed decreased quercetin derivatives due to various cooking methods in fresh-cut potato tubers. The total flavonoid contents were decreased from 7.1 to 4.0 mg/100 g FW with boiling, with steaming to 4.0, with microwaving to 3.1, and with frying to 3.3 mg/100 g of fresh weight. According to Blessington et al. [54], the contents of rutin and quercetin dehydrates were increased due to storage at 4 °C to 20 °C. The flavonoid contents were reduced after boiling, microwaving, and baking by 27%, 47%, and 52%, respectively. Therefore, the loss in flavonoid contents was more in the case of baking and microwaving [31]. More research is needed to identify the individual and total flavonoids as affected by various cooking methods.

### 2.3. Anthocyanins

The color of pigmented varieties is due to the presence of additional specific phenolic molecules, called anthocyanins. Anthocyanins in colored tubers contain mostly pelargonidin and petunidin [16]. The four major anthocyanins extracted from red-fleshed potatoes were cyanidin-3-rutinoside-5-glucoside, petunidin-3-rutinoside-5-glucoside, pelargonidin-3-rutinoside-5-glucoside, and peonidin-3-rutinoside-5-glucoside, with petunidin and peonidin glycosides being the most predominant. [14]. In purple-fleshed potato accessions, the predominant anthocyanin is petunidin-3-(coumaroyl) rutinoside-5-glucoside, representing 37–78% of the total anthocyanins [35]. In potatoes, the contents of anthocyanin ranged from 0.274 to 17.253 mg/100 g FW [49]. The contents of anthocyanins in purple potatoes ranged from 5.5 to 17.1 mg/100 g FW, and in red potato ranged from 6.9 to 35 mg/100 g FW [50]. A higher level of anthocyanins was found in an Andean cultivar with dark purple flesh for about 16.33 mg/g DW [45]. Another study demonstrates the existence of anthocyanins in purple and red potato tubers ranging from 253–2357 mg/100 g fresh weight [47], but the contents of anthocyanin might be destroyed by long-time exposure to high temperatures and overcooking; for example, frying results in 40–60% loss in anthocyanin contents in red potato cultivars, while a 50–80% loss in purple potato cultivars [3,32]. Processing methods such as the thermal processing method degrade anthocyanin contents [57]. Anthocyanin contents are also degraded due to the potato chip production process. Blanching and soaking, as commonly used commercial and domestic processing methods, result in the leaching of anthocyanin in the water because anthocyanin is highly soluble in water [58]. Studies report that the anthocyanin contents were decreased in purple-fleshed potatoes after cooking. In contrast, various cooking methods such as frying, baking, boiling, microwaving, and steaming reduced the level of anthocyanin in different potato cultivars by 83.15%, 25.67%, 14.66%, 14.01%, and 7.45%, respectively [59]. The anthocyanin content in five potato cultivars was decreased significantly after various cooking methods, but boiling increased the anthocyanin level as compared to raw potatoes. The retention of anthocyanins may take place due to the transfer of components between the oil and potato, especially during frying [56,60]. Higher anthocyanins were reported in color potatoes after cooking, while a maximum loss in anthocyanin content was found after frying due to the thermal degradation of anthocyanin, however steaming seemed to retain the anthocyanin level [61]. The contents of anthocyanins were reduced by 38–70% in red- and purple-colored potatoes after frying [32] (Table 2). Anthocyanin and carotenoids present in potatoes have a higher recovery rate after cooking as compared to other polyphenols [35]. The stability of anthocyanin is affected by pH, atmospheric condition, and temperature because it is a very unstable compound and sensitive to heat; accordingly, various cooking methods such as baking, boiling, and frying can degrade its contents.

A study reported a significant increase of anthocyanin after microwaving (459 ± 66 mg cyanindin-3-glucoside (C-3-g)/kg FW), boiling (438 ± 6 mg C-3-g/kg FW), and steaming (431 ± 18 mg C-3-g/kg FW) as compared to raw purple potatoes with 219 ± 17 mg C-3-g/kg FW. Because of the disruption of the cells, the anthocyanin content was increased after cooking [12]. Blessington et al. [54] also observed an increase in anthocyanin contents after microwaving (47.48%), frying (46.12%), and baking (36.36%). According to Lachman et al. [34], the concentration of anthocyanins in tubers was not disturbed by various cooking methods such as boiling, microwaving, and baking. The anthocyanin content found in microwaved, steamed, and boiled purple potatoes was two times higher than in raw potatoes. In contrast, no loss in anthocyanin content was found in purple potatoes. Therefore, the cooked purple potatoes have a higher anthocyanin content than raw potatoes. The increase in anthocyanins might be due to deactivation of polyphenol oxidase by thermal treatment; the enzyme degrades the anthocyanins and other polyphenols, and so its deactivation results in less anthocyanins degraded [7,12]. Overcooking can destroy polyphenols, especially anthocyanins. Therefore, a low temperature is needed during boiling, and a higher temperature for baking with a low power mode is required to retain the maximum amount of health beneficial compounds [55,62].

## 3. Health Benefits of Polyphenols in Potato

**Figure 2:**
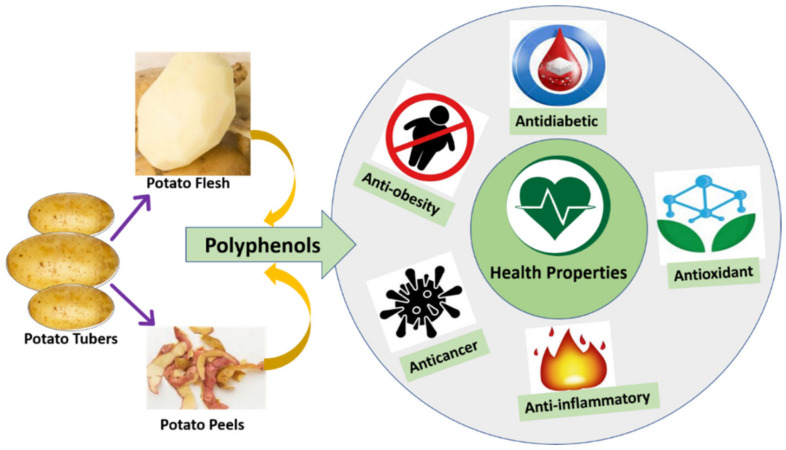
Various biological activities concerning with polyphenols extracted from potato flesh and peels.

Dietary compounds in potatoes such as polyphenols and anthocyanins are known as major contributors to the health benefits [63]. Polyphenols are considered to be health-promoting phytochemicals as they have shown in vitro antioxidant activity and have been reported to exhibit beneficial anti-bacterial, hypoglycemic, anti-viral, anti-carcinogenic, anti-inflammatory, and vasodilatory properties [64]. This section briefly describes the health properties, especially antioxidant properties, anti-obesity, anti-diabetic properties, anti-inflammatory properties, and anticarcinogenic properties, associated with polyphenols and other bioactive compounds present in potatoes (Table 3; Figure 2). All these beneficial health-promoting properties of potatoes play a significant role against some chronic diseases like cancer, type 2 diabetes mellitus, obesity, and heart diseases.

### 3.1. Antioxidant Property

**Figure 3:**
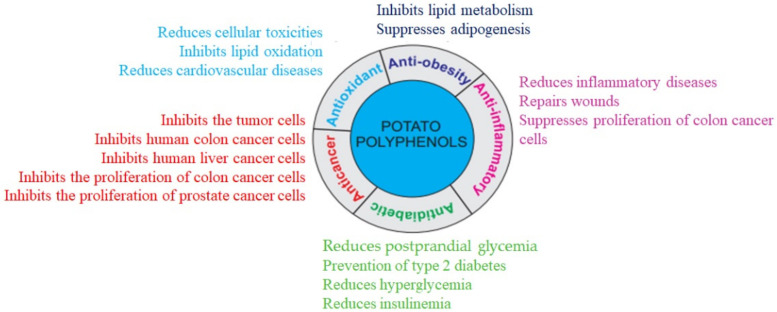
Polyphenols in potatoes represent various beneficial health properties.

Antioxidants are defined as the substances in food that significantly decrease the adverse effects of reactive species on normal physiological function in humans [65]. In the human diet, potato is one of the best sources of antioxidants, which supports the antioxidant defense to minimize cellular and tissue toxicities (Figure 3). Antioxidants function by scavenging radicals, donating electrons and hydrogen, reducing peroxides, and quenching superoxide and singlet oxygen. Because of their different reaction pathways, various methods have been developed in different systems to study free radical-scavenging antioxidant activity. For example, 2,2-diphenylpicryl-1-hydrazyl (DPPH), 2,2-azobis-3-ethyl-benzothiazoline-6-sulfonate (ABTS), oxygen radical absorbance capacity (ORAC), and ferric reducing antioxidant power (FRAP) assays are extensively used as in vitro tests for estimating antioxidant potential [66].

The antioxidants in potatoes are mainly hydrophilic (polyphenols, ascorbic acid, anthocyanins, and flavanols). Chlorogenic acid, gallic acid, caffeic acid, and catechin are significant contributors of antioxidant activity in yellow-fleshed potatoes, while chlorogenic acids and anthocyanins are the major contributors in red and purple-fleshed potatoes. Chlorogenic acid, as the most abundant potato polyphenol, inhibits lipid oxidation (Figure 3). Boiled potatoes with purple flesh have antioxidant activity ranging from 4017–17,304 µg Trolox Equivalent/g FW as determined by ABTS antioxidant capacity, and ranging from 2369–9754 µg Trolox Equivalent/g FW as determined by DPPH antioxidant assay [47]. Highly positive correlations between the total phenolic contents and antioxidant activity have been demonstrated, in which potato phenolic compounds were the main contributors to antioxidant property. The antioxidant activities differ in potatoes with different colors, while purple potato tubers were reported to have a higher level of antioxidant property as compared to others [3]. Pigmented red- and purple-flesh potatoes contain two to three times more antioxidants than white-flesh potatoes. The antioxidant properties of pigmented potatoes are accounted for by the presence of polyphenols, specifically anthocyanins, phenolic acids, and carotenoids [67]. Potato peel and neighboring tissues also contain about 50% of the polyphenols, while its amount slowly and gradually decreases towards the center of the potato tubers. Anthocyanin extracted from red potato peels has stronger antioxidant activity compared to brown potato peels extract [68]. The peels of young potato tubers are a good source of polyphenols, especially chlorogenic acid and gallic acid with excellent antioxidant activity as compared to mature potato peels [69,70]. The extract of potato peels can be used as a natural antioxidant to suppress lipid oxidation for the production of fats, oils, and other dietary products [71].

Colored potatoes had higher total phenolic content (TPC) than yellow- and white-flesh potatoes, which resulted in higher antioxidant capacity than yellow and white potatoes [20]. As such, potatoes with purple flesh showed high antioxidant, antimicrobial and antiproliferative effects against various cancer cells [72]. Indeed, 300 g flakes of purple potatoes potentially decreased the thiobarbituric acid-reactive substance level in liver and serum and increased the antioxidant enzymes activities in the liver as compared to white potatoes when fed to hypercholesterolemic rats [44].

### 3.2. Anti-Obesity Property

Whether consumption of potatoes can cause obesity is under debate. Potatoes contain a very low amount of fat content (about 0.1%) which has no negative nutritional effects [73]. Potatoes are known as a high-calorie food compared to other vegetables because of their high carbohydrate content [74]. Most developing countries consume potatoes in very large quantities. The consumption of deep-fried food on a regular basis can lead to the risks of obesity, overweight, and various other diseases [75]. Deep-fried potatoes are also associated with these diseases in both men and women [63].

Many studies reported the beneficial health properties of polyphenols in potato in human cell culture [76], such as anti-obesity [77], antidiabetic, hypocholesterolemia [78], and anti-inflammatory effects [79]. The presence of a comparatively high quantity of phytonutrients with bioactivities might be helpful in the prevention of chronic diseases, but is massively underestimated in the case of potatoes [80]. Chlorogenic acid is reported as an anti-obesity agent [77]. Supplementation of chlorogenic acid may significantly reduce the triglyceride level, while increasing the plasma adiponectin level, as compared to the high-fat control diet group [81].

Research recommends that the consumption of French fries leads to increased risks of type 2 diabetes and obesity [82]. However, a study suggests the anti-obesity property of purple potato on rats. The rats were fed a high-fat diet along with potato ethanolic extract, which inhibits lipid metabolism through down-regulation of the expression of p38 mitogen-activated kinase (MAPK) along with uncoupled protein-3 (UPC-3), and thus showed high anti-obesity properties [83]. Another 10-week study was conducted in which both male and female mice were fed with a high-fat diet with and without polyphenolic-rich extract of potato. The polyphenolic-rich potato extracts resulted in the reduction of weight and adiposity in both sexes of mice by 63.2% in males and females, as compared to extracts prepared without polyphenols. [84].

### 3.3. Antidiabetic Property

Potato has a glycemic index (GI) ranging from 53–103 [85]. Consumption of high GI food may lead to an increase in the risks of emerging type 2 diabetes, obesity, and cardiovascular diseases [86]. However, a recent cohort study provide evidence that moderate intake of total and boiled potato may be associated with a decreased risk for incident diabetes, but there was no association between fried potato and the risk for diabetes [87]. The most abundantly found phenolic acid in potato, chlorogenic acid, helps to slow down the glucose output into the blood and results in the reduction of GI. Moreover, potato peels significantly reduced hyperglycemia, overall food intake, and oxidative stress in diabetic rats because of their high level of polyphenols [7]. The chips of purple-fleshed potatoes reduce blood glucose in animal studies models and act as anti-diabetic agents [88]. Experimental data suggest a positive association between type 2 diabetes and potato consumption [89]. Purple polyphenol-rich potatoes significantly reduced insulinemia and postprandial glycemia compared with yellow potatoes in a randomized cross-over trial on 17 healthy volunteer males who consumed potatoes with yellow flesh with or without purple potato extract [90].

The intake of purple-fleshed potatoes may prevent diabetes by improving serum insulin levels as was shown in diabetic rates [91]. A study evaluated the GI of white, yellow, red, and purple potatoes in healthy adults: the GI of red potatoes was 78, purple potatoes were 77, yellow potatoes were 81, while white potatoes were recorded as 93 [67]. The polyphenols in red potatoes were 190 mg/100 g DW, in purple potatoes were 234 mg/100 g DW, in yellow potatoes were 108 mg/100 g DW, while in white potatoes were 82 mg/100 g DW. These findings suggest that colored potatoes with high polyphenols have low values of GI which is basically due to the inhibition of α-glycosidase with the help of anthocyanins [67]. Additionally, highly pigmented potatoes tend to reduce glucose responses and GI values compared to white or yellow varieties [23,67].

### 3.4. Anti-Inflammatory Properties

Inflammation is the process through which the immune system recognizes and removes harmful and foreign stimuli [92], while the anti-inflammatory process reverses the tissue homeostasis to normality [93]. Various anti-inflammatory compounds have been found in potatoes, including anthocyanins that contribute to reducing inflammatory bowel syndrome and various other chronic diseases related to gut health [79,94]. Polyphenols extracted from potato and onion inhibit the lipopolysaccharide (LPS) stimulated cyclooxygenase-2 (COX-2) expression compared to the non-steroidal anti-inflammatory drug (NSAID) [95], which indicates that polyphenols extracted from potato can help as a natural source of anti-inflammatory substances. The anti-inflammatory and antioxidant properties of purple potatoes were higher than that of white potatoes. In humans, purple potatoes reduce the levels of plasma high-sensitivity C-reactive protein and significantly increase the wound repair in the intestinal epithelial cells (IEC-6) [96,97]. The consumption of potatoes significantly reduced the rate of current wheeze in Colombian children (6–7 years old) [98]. In a randomized study, cooked potatoes were consumed by healthy men (18 to 40 years of age) once daily for six weeks. The colored potatoes significantly reduced inflammation as compared to non-colored potatoes because colored potatoes have higher phenolic acid and anthocyanin content [99]. The oral administration of potato extract to mice at doses of 100 and 200 mg/kg produced anti-nociceptive effects in response to formalin-induced pain licking and hot plate-induced pain. In mice, the potato ethanolic extract also inhibited formalin-induced and carrageenan inflammation, which indicates that ethanolic extract of potato tubers relieves inflammatory pain [100]. The derivatives of anthocyanin inhibited the pro-inflammatory cytokine secretion [101], while anthocyanins extracted from purple potatoes may enhance intestinal epithelial cell differentiation [102]. Anthocyanins affect colonic-systemic inflammation because of direct contact with the gut and their higher concentration; however, anthocyanins are present in small amounts in systemic circulation [101]. Bacteria associated with gut health play a vital role in the spread of various kinds of inflammatory diseases. An increased number of *Clostridium* species, such as Bacteroidaceae and Enterobacteriaceae, were observed in a mice model of colitis in comparison to control mice. Anthocyanins help to reduce pathogenic bacteria in the gut, while promoting health beneficial bacteria; for example, anthocyanins decrease gut luminal LPS and improves the growth of *Lactobacillus* and *Bifidobacterium* species [79]. In comparison with antibiotic drugs, chloramphenicol extract of purple potato showed the highest inhibition against *E. coli* bacteria, which is associated with colonic inflammation [96]. The importance and huge consumption in human diets make potatoes a good source of anti-inflammatory compounds.

### 3.5. Anticancer Properties

Potato antioxidants such as phenolic acid and anthocyanins have anticarcinogenic effects [79]. Studies suggest that the consumption of potatoes may reduce the risk of pancreatic cancer, colorectal cancer, and colon cancer [103]. In Caco-2 cells, the anthocyanins extracted from colored potatoes helps to reduce interleukins IL6, IL-8, 1β, and (TNF)-α (tumor necrosis factor) induced by H_2_O_2_ [104]. After digestion of cooked purple-fleshed tubers in a dynamic human gastrointestinal model, the colonic fecal water showed potent effects on the cytotoxicity and cell viability of colonic tumor cells [105]. Anthocyanins extracted from potato with purple flesh suppress the proliferation and apoptosis of colon cancer cells as compared to potatoes with white and yellow flesh [106], while antiproliferative and antioxidant properties of anthocyanins act against breast cancer cells (MDA-MB-231 and MCF-7), and colon cancer cells (SW48 and CaCo-2) after GI digestion [72]. Anthocyanins extracted from colored potatoes induced apoptosis of human stomach cancer cells (KATO III) [107].

Polyphenols, especially anthocyanins, reveal chemotherapeutical activity that makes them suitable for cancer treatment by the modulation of a variety of molecular targets. Anthocyanin-rich purple-fleshed potatoes reduce colon tumorigenesis through the elimination of colon cancer stem cells by suppression of Wnt/β catenin signaling (Table 3) [108]. Five potato lines were assessed for total phenolics, antioxidant activity, anthocyanins, chlorogenic acid, and anticancer activity. Results indicate that potatoes with higher polyphenol content have maximum antioxidant activity and good inhibitory function against the proliferation of cancer cells. This inhibitory effect varies among various tested potato lines. Phenolic acids such as chlorogenic acid significantly inhibit prostate cancer and proliferation of colon cancer, and inhibit human colon and liver cancer cells [109]. Extract of purple-fleshed potato was also effective against colon tumorigenesis via the elimination of colon cancer stem cells, and steamed red and purple potatoes inhibited the growth of benzopyrene-induced stomach cancer in mice [108].

Anthocyanin and the extract of colored potato help to suppress lymph node carcinoma of the prostate and proliferation of prostate cancer cells and induce apoptosis; gallic acid extracted from potatoes also decreases prostate cancer and induces apoptosis [110]. Anthocyanins of potato extracts were found to be more cytotoxic against prostate cancer cells [79]. The extract of four potato cultivars up-regulated the cyclin-dependent kinase inhibitor p27, and thus was effective against prostate cancer cells (PC-3 and LNCaP) (Table 3). Red- and purple-fleshed potatoes have an inhibitory effect on stomach cancer [111]. Numerous studies demonstrate that anthocyanin is effective against various diseases like dementia by enhancing cognition and colorectal cancer in mice [112,113].

## 4. Conclusions

Potato is extensively consumed worldwide, but the pigmented varieties may have a particular advantage over non-pigmented varieties due to high polyphenolic contents. Potato polyphenols such as phenolic acids, flavonoids, and anthocyanins are found both in the peel and flesh. Chlorogenic acid, ferulic acid, and caffeic acid are reported as major phenolic acids found in potatoes. Potato polyphenol content varies depending on genotype, growing condition, cooking method. Potato polyphenols play a vital role in various biological activities, in particular, antioxidant, antibacterial, anticarcinogenic, antidiabetic and anticancer activities. Information based on the health benefits and various biological activities of polyphenols might aid the design of better food products, and would also be helpful in various industries, such as the food, cosmeceutical, and pharmaceutical industries.

## Figures and Tables

**Table 1:** Diverse range of polyphenols in potatoes.

Polyphenols	Polyphenols	Range (mg/100 g)	References
Phenolic acids	Chlorogenic acid	3.49–73.4	[18]
		0.77–7.98	[21]
		41.86–141.58	[37]
		17.3–148.1	[38]
		2.14–2.92	[36]
		2.06–79.91	[20]
		314.9–2401	[34]
		43–953	[15]
		47–283	[39]
		23.2–61.4	[24]
		1.4–12.1	[40]
		0.9–27.0	[41]
	Cryptochlorogenic acid	8.0–59.0	[36]
		0.09–1.50	[21]
		3.1–163.3	[38]
	Caffeic acid	0.46–3.21	[18]
		0.1–0.2	[42]
		1.1–172.4	[38]
		5.0–50.0	[39]
		20.3	[43]
	Ferulic acid	0.6–9.0	[39]
		0–3.9	[38]
	Coumaric acid	0–1.6	[38]
		0–9.2	[39]
	Neochlorogenic acid	3.0–11.0	[36]
		49.2–91.2	[38]
		2.9–9.9	[42]
		2.1–7.13	[24]
	Vanillic acid	0–22.4	[38]
	Protocatechuic acid	0–7.6	[38]
	*p*-hydroxybenzoic acid	0–7.8	[38]
	Gallic acid	0–1.0	[38]
		414	[44]
Flavonoids	Quercetin	0.025	[42]
		0–4.78	[45]
	Kaempferol	0.5–1.7	[36]
		0–5.68	[45]
	Catechin	0–204	[26]
		0–1.4	[46]
		0–1.5	[38]
		43.0–204.0	[39]
		29–211	[44]
Anthocyanins	Anthocyanins	21.0–109.0	[32]
		10–39	[16]
		8.2–152.7	[14]
		0–87.0	[44]
		16.33	[45]
		418.0	[47]
		0.24–1.46	[48]
		0.274–17.253	[49]
		5.5–17.1	[50]
		5.52–34.96	[10]
		18.6–22.9	[15]

Concentrations of reported polyphenols expressed as mg/100 g FW.

**Table 2:** Effect of cooking on the polyphenol content of potatoes.

Cooking Method	Phenolic Acids	Flavonoids	Anthocyanins	References
Frying	0–59.25% ↓	-	38–70% ↓	[32]
Boiling	66.02% ↓	-	100.00% ↑	[12]
Baking	18.18% ↓	-	103.19% ↑	
Microwaving	35.40% ↓	-	209.58% ↑	
Steaming	62.20% ↓	-	196.80% ↑	
Boiling	18.47–69.66% ↑	-	0–23.15% ↓	[47]
Microwaving	-	-	129.79–847.83% ↑	[34]
Baking	-	-	85.62–557.14% ↑	
Boiling	ND	-	16.25–20.04% ↓	[55]
Microwaving	ND	-	18.01–29.73% ↓	
Boiling	6.37–52.60% ↓	-	-	[56]
Boiling	3.32% ↓	-	-	[54]
Baking	37.27% ↑	-	36.36% ↑	
Microwaving	49.08% ↑	-	47.48% ↑	
Frying	46.86% ↑	-	46.12% ↑	
Boiling	27.16–71.26% ↑	147% ↑	-	[36]
Baking	49.04–119.76% ↑	178.3% ↑	-	
Microwaving	2.35–52.00% ↑	-	-	
Steaming	15.63–93.41% ↑	129.7% ↑	-	
Boiling	81.4% ↑	-	-	[30]
Microwaving	80.81% ↑	-	-	
Steaming	22.8% ↑	-	-	
Boiling	-	-	14.66% ↓	[7]
Frying	-	-	83.02% ↓	
Baking	-	-	25.67% ↓	
Microwaving	-	-	14.01% ↓	
Steaming	-	-	7.45% ↓	
boiling	44% ↓	27% ↓	-	[31]
Microwaving	52% ↓	47% ↓	-	
Baking	53% ↓	52% ↓	-	
Boiling	-	56.3% ↓	-	[29]
Steaming	-	56.3% ↓	-	
Microwaving	-	43.6% ↓	-	
Frying	-	46.47% ↓	-	

↓ Decreased; ↑ Increased; - no data; ND: no difference.

**Table 3:** Health properties and physiological effects of potato polyphenols.

Active Ingredient	Biological Effects	Health Properties	References
Phenolic compounds	Up-regulate expression of cellular antioxidant enzymes; prevent oxidative damage to DNA and other biomolecules; inhibit the growth of few pathogenic fungi	Antioxidant	[63,114,115]
	Target stem cells of cancer; prevent the proliferation of cancer cells; cytotoxic to prostate cancer; inhibits prostate cancer, the proliferation of colon cancer; inhibits human colon and liver cancer cells	Anticancer	[108,109,111,116,117,118]
	Reduce inflammation and edema volume increment	Anti-inflammatory	[119]
	Reduce postprandial glycemic response; inhibit hepatic glucose-6-phosphatase; reduce gut glucose absorption; reduce oxidative stress and overall food intake	Antidiabetic	[80,84,90,120]
	Suppress adipogenesis; inhibit lipid metabolism through down-regulation of expression of p38 mitogen-activated protein kinase (MAPK) and uncoupling protein 3 (UCP-3)	Anti-obesity	[83,84]
Anthocyanins	Reduce systolic and diastolic blood pressure; cytotoxic to prostate cancer; suppress (LNCaP) lymph node carcinoma of prostate and (PC3) prostate cancer cells	Antihypertensive	[80,110,111,121]
	Improve insulin sensitivity; reduce the values of GI by inhibiting α-glycosidase	Antidiabetic	[67,122]
	Up-regulate the cellular antioxidant enzyme expression; inhibit lipid oxidation; protect DNA from oxidation damage	Antioxidant	[44,99,123]
	Reduce 8-hydroxydeoxyguanosine (8-OHdG); interleukin6 (IL-6) and plasma concentration of CRP	Anti-inflammatory	[99]
	Cytotoxic effect against various cancer cells; suppress the elevation and proliferation of colon tumor cells via suppression of Wnt/β-catenin signaling; inhibit metastasis-related proteins MMP- 2 and MMP-9 expression and caspase 3-dependent mitochondrial apoptosis; reduce interleukins IL6, IL-8, 1β, and (TNF)-α (tumor necrosis factor) induced by H_2_O_2_; suppress the proliferation and apoptosis of colon cancer cells, antiproliferative activity against breast cancer cells (MDA-MB-231 and MCF-7) and colon cancer cells (SW48 and CaCo-2); induce nuclear uptake of proapoptotic Endo G and AIF protein and induce mitochondrial release	Anticancer	[72,76,104,106,107,108,111,124]
